# Alkaline ceramidase 2 is a novel direct target of p53 and induces autophagy and apoptosis through ROS generation

**DOI:** 10.1038/srep44573

**Published:** 2017-03-15

**Authors:** Yitao Wang, Chunxue Zhang, Yuelei Jin, Wang, Qing He, Zhu Liu, Qing Ai, Yunlong Lei, Yi Li, Fangzhou Song, Youquan Bu

**Affiliations:** 1 Department of Biochemistry and Molecular Biology, Chongqing Medical University, Chongqing 400016, China; 2 Molecular Medicine and Cancer Research Center, Chongqing Medical University, Chongqing 400016, China

## Abstract

ACER2 is a critical sphingolipid metabolizing enzyme, and has been shown to be remarkably up-regulated following various stimuli such as DNA damage. However, the transcriptional regulatory mechanism of ACER2 gene and its potential role in the regulation of autophagy remain unknown. In this study, we have for the first time identified the human ACER2 gene promoter, and found that human ACER2 transcription is directly regulated by p53 and ACER2 is implicated in the induction of autophagy as well as apoptosis. A series of luciferase reporter assay demonstrated that ACER2 major promoter is located within its first intron where the consensus p53-binding sites exist. Consistently, forced expression of p53 significantly stimulated ACER2 transcription. Notably, p53-mediated autophagy and apoptosis were markedly enhanced by ACER2. Depletion of the essential autophagy gene ATG5 revealed that ACER2-induced autophagy facilitates its effect on apoptosis. Further studies clearly showed that ACER2-mediated autophagy and apoptosis are accompanied by ROS generation. In summary, our present study strongly suggests that ACER2 plays a pivotal role in p53-induced autophagy and apoptosis, and thus might serve as a novel and attractive molecular target for cancer treatment.

Tumor suppressor p53 plays a crucial role in the regulation of stress response and tumor development^1^,^2^,^3^,^4^,^5^. p53 acts as nuclear transcription factor to regulate a variety of its target genes implicated in diverse cellular processes including DNA repair, cell cycle arrest, apoptosis, autophagy, senescence, angiogenesis and migration^6^,^7^,^8^,^9^,^10^. A growing body of recent evidence suggests that, in addition to stress response, p53 also modulates cellular metabolism such as glycolysis, oxidative phosphorylation, fatty acid metabolism, amino acid metabolism and reactive oxygen species through transactivating metabolic enzyme-related genes^11^,^12^. Indeed, it has been shown that several genes encoding important metabolic enzymes are the direct transcriptional targets of p53 including TP53-induced glycolysis and apoptosis regulator (TIGAR), glutaminase 2 (GLS2) and cytochrome oxidase 2 (Sco2)^13^,^14^,^15^. As expected, p53 has an ability to impair the oncogenic metabolic reprogramming required for cancer cell growth and survival^11^,^12^.

Sphingolipids are membrane lipids that are ubiquitously expressed in all eukaryotic cells. In addition to their structural roles in membrane biology, sphingolipids act as bioactive signaling molecules within cells. For example, metabolites of sphingolipid such as ceramide, sphingosine and sphingosine-1-phosphate (S1P) have been shown to participate in various cellular processes including proliferation, differentiation, adhesion, migration, apoptosis and autophagy^16^,^17^,^18^,^19^,^20^,^21^,^22^. Ceramidases represent a family of sphingolipid-metabolizing enzymes, which catalyze the hydrolysis of ceramides to generate sphingosine. To date, five distinct human ceramidases have been identified including acid ceramidase (AC), neutral ceramidase (NC), alkaline ceramidase 1 (ACER1), alkaline ceramidase 2 (ACER2) and alkaline ceramidase 3 (ACER3)^23^. Among these five ceramidases, ACER2 is ubiquitously expressed at low level in various normal tissues except placenta, and remarkably up-regulated in response to various cellular stimuli such as DNA damage and serum starvation^23^,^24^,^25^,^26^,^27^,^28^. Recently, it has been shown that DNA damage-mediated up-regulation of ACER2 promotes apoptosis^28^. Of note, dysregulation of ACER2 has been observed in several types of cancers. However, the transcriptional regulatory mechanism of ACER2 gene following cellular stimuli and its potential role in the regulation of certain important biological processes such as autophogy and tumorigenesis remain largely unknown^24^,^29^.

In the present study, we have identified ACER2 promoter and also found that ACER2 is a direct transcriptional target gene of p53. Furthermore, we have demonstrated that ACER2 is required for the induction of autophagy and apoptosis through the enhancement of ROS generation. Together, our present findings strongly suggest that ACER2 which is tightly implicated in sphingolipid metabolism plays an important role in the regulation of p53-dependent autophagy and apoptosis.

## Results

### Gene organization and chromatin state of ACER2 gene locus

**Figure 1:**
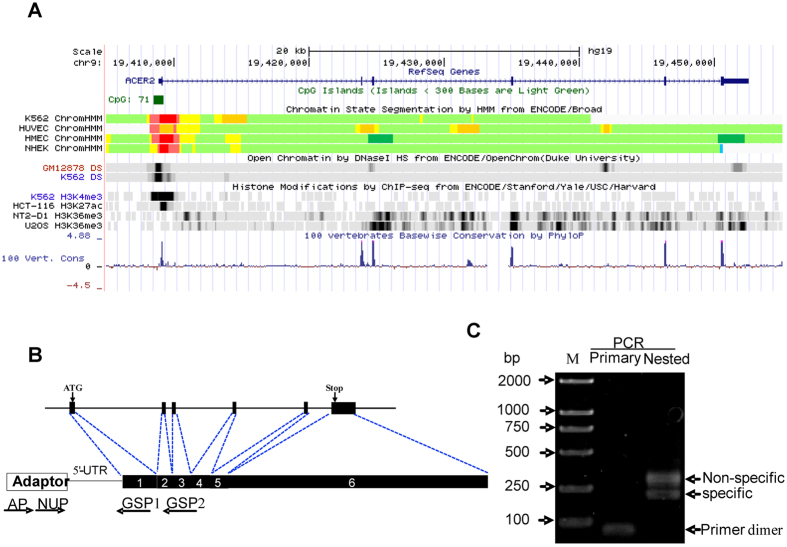
Chromatin state and 5′-RACE analysis of Human ACER2 gene. (**A**) Chromatin state annotation of human ACER2 gene locus. The genomic region of ACER2 (chr9:19405000-19455000, human species genomic assembly version, GRCh37/hg19) was retrieved and is schematically represented with the indicated tracks (https://genome.ucsc.edu/). The chromatin state segmentation is displayed as active promoter (bright red), strong enhancer (orange), insulator (blue), transcriptional elongation (dark green) and weakly transcribed region (light green). (**B**) Schematic representation of human ACER2 gene organization and 5′-RACE primer design. Exons and introns are indicated by filled boxes and thin lines, respectively. The translation start codon (ATG) and the translation stop codon are indicated by arrows. (**C**) 5′-RACE analysis. 5′-RACE analysis was conducted using human testis total RNA. Primary and nested PCR were performed using AP/GSP2 and NUP/GSP1, respectively. PCR products were analyzed on 1.0% agarose gel electrophoresis, purified for cloning and subsequently verified by sequencing. Lane M: DL2000 size markers; Lane 1: Primary PCR products; Lane 2: Nested PCR products.

To analyze the genomic organization and chromatin state of human ACER2 gene, we have employed UCSC genome browser (https://genome.ucsc.edu/). As shown in Fig. 1A, ACER2 gene is mapped at chromosome 9p22 and composed of six exons and five introns. According to ENCODE histone modification data, the transcription elongation hallmark of H3K36me3 accumulated within ACER2 gene body. Consistent with this finding, ChromHMM chromatin state segmentation data indicated that ACER2 gene might be actively transcribed. Of note, the first entire exon and the 5′-region of the first intron of ACER2 gene contained a classic CpG island with high DNase I hypersensitivity and H3K4me3 (a hallmark of transcription initiation), suggesting that ACER2 gene promoter might be present within the first exon and/or 5′-part of the first intron of ACER2 gene.

### Identification of the transcription start site(s) of ACER2 gene

To identify the transcription initiation site(s) of ACER2 gene, we have employed SMART RACE assay system using two reverse primers (GSP1 and GSP2) and adapter primers (NUP and AP) (Fig. 1B). As shown in Fig. 1C, the primary PCR (AP/GSP2) failed to amplify any products, whereas the nested PCR (NUP/GSP1) generated two distinct products. Based on the subsequent cloning and sequencing of the above two PCR products, the shorter fragment (191 bp in length without RACE adapter primer) was specific to ACER2 gene. The first base of this PCR product (G, guanine) was mapped at 76 bp upstream of ACER2 start codon, which might be the transcription initiation site of ACER2 gene (+1).

### Identification of the ACER2 promoter region

**Figure 2:**
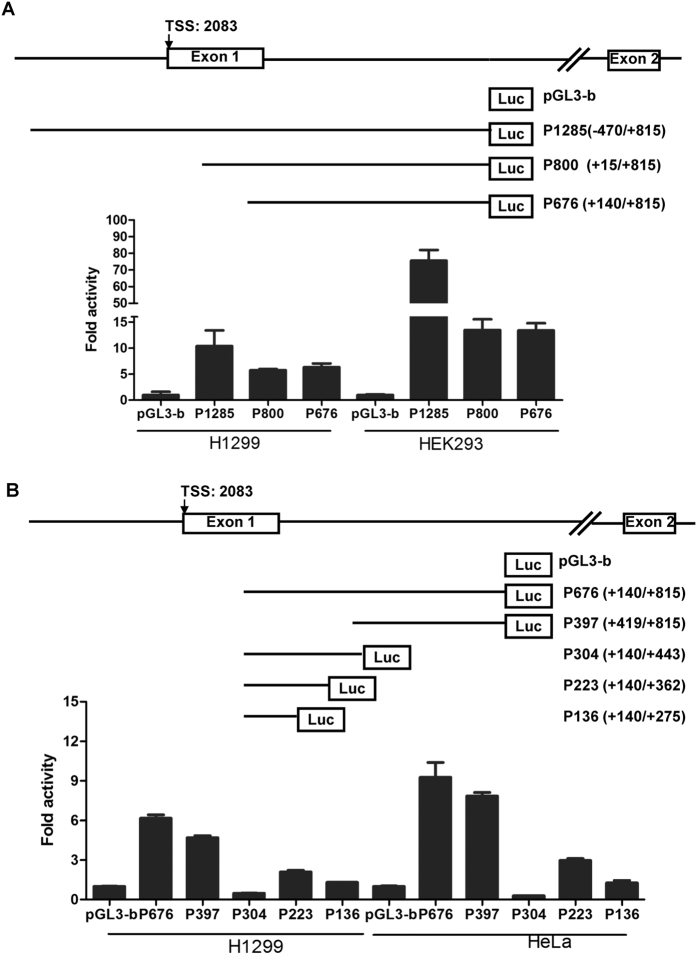
Identification of ACER2 promoter region. (**A**) ACER2 genomic region between +140 and +815 contains a strong promoter activity. A schematic diagram of ACER2 gene promoter reporter constructs (upper). The positions relative to the major ACER2 transcription start site (+1) are indicated. Exons and introns of ACER2 are shown by open boxes and thin lines, respectively. For luciferase reporter assay, H1299 (left) and HEK293 (right) cells were transiently co-transfected in triplicate in 12-well plates with the indicated luciferase reporter constructs together with Renilla luciferase reporter plasmid (pRL-TK) by using Lipofectamine 2000 transfection reagent. Forty-eight hours after transfection, firefly and Renilla luciferase activities were measured by Dual Luciferase Assay System (Promega). Data obtained from a representative of at least three independent experiments were shown as fold induction compared to the activity of cells transfected with the empty pGL3-basic vector (lower). (**B**) The major ACER2 promoter exists within its first intron (+419/+815). A schematic diagram of ACER2 gene promoter reporter constructs (upper). For luciferase reporter assay, H1299 and HeLa cells were transiently co-transfected with the indicated luciferase reporter constructs along with pRL-TK. Forty-eight hours after transfection, firefly and Renilla luciferase activities were measured and analyzed as in Fig. 2A (lower).

To identify the potential proximal promoter region(s) of ACER2 gene, we generated three luciferase reporter constructs containing the indicated genomic fragments of ACER2 gene such as P1285 (−470/+815), P800 (+15/+815) and P676 (+140/+815) (Fig. 2A). The results obtained from the chromatin state and transcription start site analyses (Fig. 1) prompted us to generate P1285 (−470/+815) carrying the 5′-upstream region, first exon and 5′-part of the first intron of ACER2 gene. The indicated luciferase reporter constructs were introduced into H1299 and HEK293 cells and their luciferase activities were measured. As shown in Fig. 2A, a significant increase in luciferase activities arising from each of three constructs [P1285 (−470/+815), P800 (+15/+815) and P676 (+140/+815)] was detectable as compared to that of the empty pGL3-basic plasmid, suggesting that a genomic region from +140 to +815 of ACER2 gene has a strong promoter activity.

To further delineate ACER2 gene promoter, we have generated the additional four luciferase reporter constructs including P397 (+419/+815), P304 (+140/+443), P223 (+140/+362) and P130 (+140/+275), based on their parental P676 (+140/+815) (Fig. 2B). Luciferase reporter assay demonstrated that luciferase activity driven from P397 (+419/+815) is comparable to that from the parental P676 (+140/+815), whereas P304 (+140/+443) fails to drive luciferase reporter gene expression, indicating a genomic region from +419 to +815 of ACER2 gene contains an obvious promoter activity. Intriguingly, P223 (+140/+362) had a lower but considerable promoter activity relative to pGL3-basic vector, suggesting that the genomic region from +140 to +362 contains an alternative minor promoter for ACER2 gene.

Taken together, we concluded that the major ACER2 promoter exists within the first intron spanning from +419 to +815.

### Sequence and homology analysis of the ACER2 promoter

**Figure 3:**
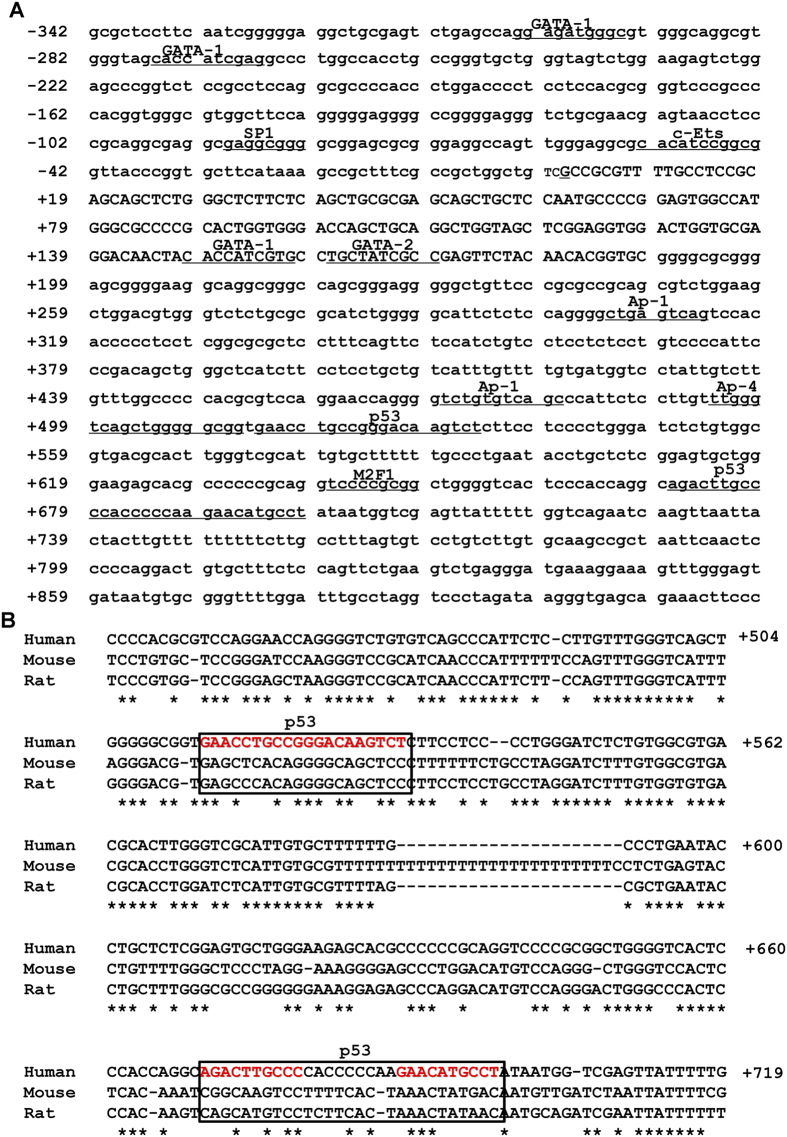
ACER2 gene promoter region contains consensus p53-binding sites. (**A**) Nucleotide sequence of human ACER2 promoter region. The potential transcription factor binding sites are underlined. Nucleotides are numbered relative to the major transcription start site of ACER2 (+1). (**B**) Sequence alignment of human, mouse and rat ACER2 gene promoters. The potential p53-binding sites are boxed.

To gain an insight into understanding the transcriptional regulatory mechanisms of ACER2 gene, the promoter region of ACER2 gene was subjected to transcription factor binding site analysis using MatInspector professional and TFSEARCH software. As shown in Fig. 3A, ACER2 gene promoter lacks the classical TATA box, but contains the classical GC box and the other putative binding sites of transcription factors such as p53, AP-1 and GATA-1. In addition, homology analysis using ClustalW2 algorithm revealed that ACER2 promoter sequence is evolutionarily conserved across various species such as human, mouse and rat (Fig. 3B). This high degree of homology among ACER2 promoter regions was comparable to that among the coding regions of ACER2. Since two potential p53-binding sites were evolutionarily well conserved among species, it is highly suggestive that p53 might directly regulate ACER2 gene transcription.

### p53 directly transactivates ACER2

**Figure 4:**
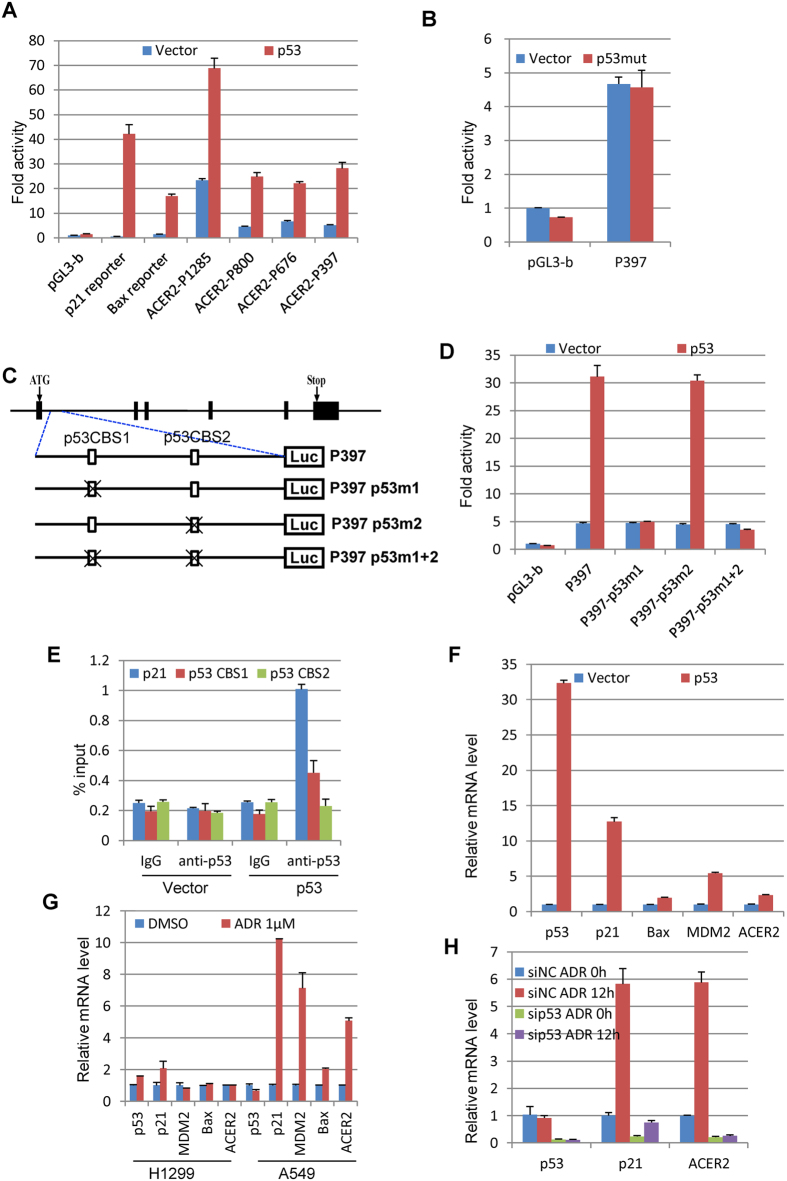
ACER2 is a direct transcriptional target of p53. (**A**) Overexpression of wild-type p53 enhances ACER2 promoter activity. p53-deficient H1299 cells were transiently co-transfected with the indicated luciferase reporter constructs and pRL-TK together with the expression plasmid for wild-type p53. Forty-eight hours after transfection, their luciferase activities were examined as in Fig. 2. (**B**) Mutant p53 has an undetectable effect on ACER2 promoter activity. H1299 cells were co-transfected with ACER2-P397 reporter construct and pRL-TK along with mutant p53 (R175H) expression plasmid. Forty-eight hours after transfection, luciferase activities were examined as in (A). (**C**) Schematic diagram of the luciferase reporter plasmids containing ACER2 promoter region with the indicated point mutations at the potential p53-binding sites (p53CBS1 and p53CBS2). Exons of ACER2 are indicated as filled boxes (upper) and the potential p53-binding sites are shown as open boxes (lower). (**D**) p53CBS1 is required for p53-dependent activation ACER2 promoter. The indicated luciferase reporter constructs were introduced into H1299 cells together with empty plasmid or with wild-type p53 expression plasmid. Forty-eight hours after transfection, their luciferase activities were determined as in (**A**). (**E**) Direct recruitment of p53 onto ACER2 promoter region in cells. H1299 cells were transfected with p53 expression plasmid or with the empty plasmid. Forty-eight hours after transfection, chromatin fragments were prepared and immunoprecipitated with p53 antibody or with control IgG. The co-precipitated DNA fragments were purified and subjected to qPCR analysis. p53-target p21 promoter was amplified as a positive control. (**F**) p53 activates the endogenous ACER2 transcription. H1299 cells were transiently transfected with the empty plasmid or with p53 expression plasmid. Forty-eight hours after transfection, total RNA was extracted and subjected to qRT-PCR with the indicated primer sets. (**G**) Adriamycin-dependent induction of the endogenous ACER2. p53-deficient H1299 cells and p53-proficient A549 cells were treated with or without 1 μM of adriamycin (ADR). Twenty-four hours after treatment, qRT-PCR was conducted. (**H**) p53 is required for DNA damage-induced ACER2 up-regulation. A549 cells were transfected with control siRNA or with p53-specific siRNA. Twenty-four hours after transfection, cells were treated with 1 μM of ADR. Twenty-four hours after treatment, qRT-PCR was conducted.

To ask whether p53 could directly transactivate ACER2 gene, p53-deficient H1299 cells were co-transfected with p53 expression plasmid together with the indicated ACER2 luciferase reporter plasmids. Luciferase reporters bearing p53-target p21 and Bax promoters were employed as positive controls. The luciferase reporter assay revealed that ectopic expression of wild-type p53 enhances luciferase activities driven by the indicated ACER2 promoters including P1285, P800, P676 and P397 (Fig. 4A). Under our experimental conditions, p21 and Bax promoters responded to the exogenous p53. In a sharp contrast to wild-type p53, mutant p53 (p53R175H) had an undetectable effect on P397 ACER2 luciferase reporter (Fig. 4B).

To verify whether p53-mediated ACER2 transactivation could be dependent on the putative p53-binding sites of ACER2 promoter, we have introduced point mutation(s) at the two putative p53-binding site(s) of ACER2 promoter and generated P397 p53m1, P397 p53m2 and P397 p53m1 + 2 luciferase reporter plasmids (Fig. 4C). H1299 cells were then co-transfected with p53 expression plasmid together with the indicated luciferase reporter plasmids. As shown in Fig. 4D, disruption of the first p53-binding site (p53CBS1) but not the second one (p53CBS2) completely impaired p53-dependent ACER2 transactivation, suggesting that the first p53-binding site is required for p53-mediated ACER2 transcriptional activation. Consistent with these results, ChIP assay demonstrated that, like p21 promoter, p53 is efficiently recruited onto the first p53-binding site within ACER2 promoter region in cells (Fig. 4E).

Next, we tested whether p53 could transactivate the endogenous ACER2 expression. As shown in Fig. 4F, ectopic expression of wild-type p53 resulted in up-regulation of the endogenous ACER2 in H1299 cells. Under our experimental conditions, p53-dependent endogenous transcriptional induction of p21, Bax and MDM2 was detectable. Then, we assessed whether ACER2 could be induced following DNA damage in a p53-dependent manner. To this end, p53-deficient H1299 and p53-proficient A549 cells were treated with 1 μM of adriamycin (ADR). Twenty-four hours after treatment, total RNA was prepared and subjected to qRT-PCR. As seen in Fig. 4G, a massive increase in the endogenous ACER2 transcription was detected in A549 cells but not in H1299 cells. Moreover, siRNA-mediated silencing of p53 in A549 cells significantly prohibited ADR-dependent transcriptional induction of ACER2 (Fig. 4H). Collectively, our results strongly suggest that ACER2 is a novel direct target gene of p53.

### ACER2 stimulates autophagy and apoptosis

**Figure 5:**
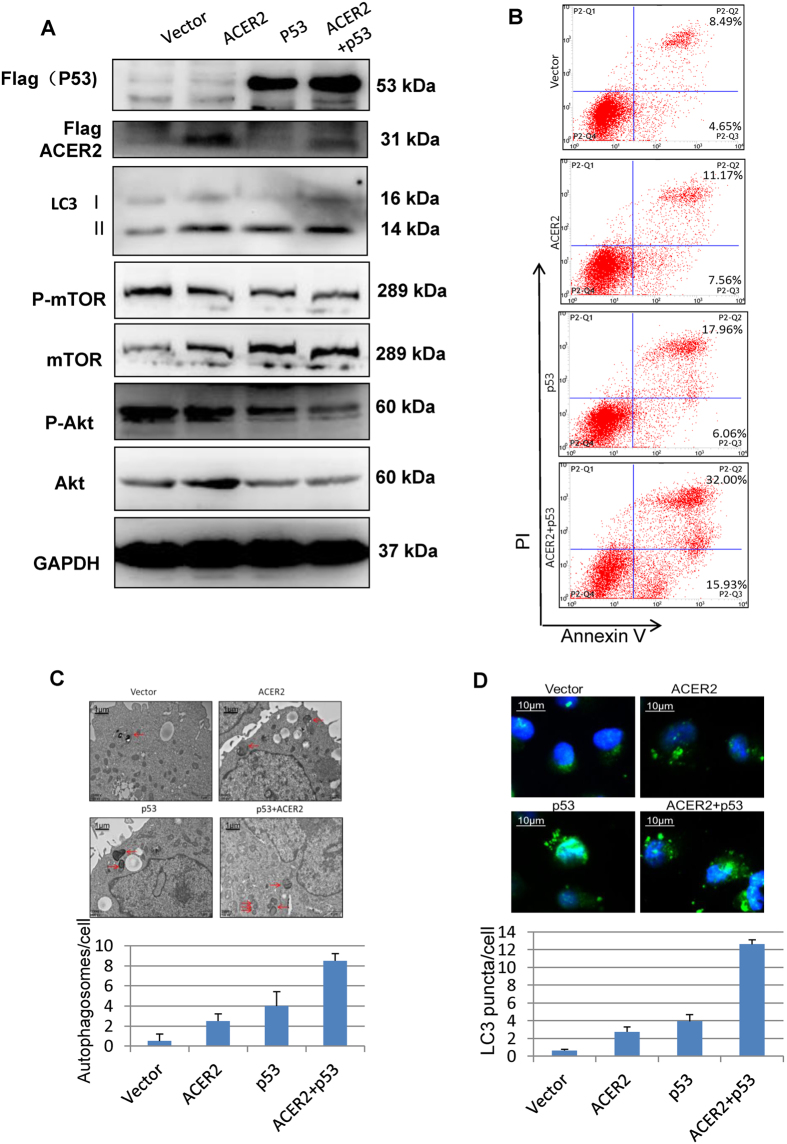
ACER2 induces apoptosis and autophagy. (**A**) Forced expression of ACER2 induces autophagy accompanied by the inhibition of mTOR-Akt pathway. H1299 cells were transiently transfected with the indicated combinations of the expression plasmids. Forty-eight hours after transfection, cell lysates were prepared and subjected to immunobloting with the indicated antibodies. (**B**) ACER2 induces apoptosis. H1299 cells were transfected as in (**A**). Forty-eight hours after transfection, cells were subjected to the flow cytometric analysis. (**C**) and (**D**) ACER2 induces autophagy. H1299 cells were transfected as in (**A**). Forty-eight hours after transfection, cells were analyzed by the transmission electron microscopy (**C**) and the indirect immunofluorescence staining with LC3 antibody (**D**).

Since p53 plays an important role in the regulation of both autophagy and apoptosis, we next asked whether ACER2 could induce autophagy and/or apoptosis in a p53-dependent manner. For this purpose, H1299 cells were transfected with the indicated combinations of the expression plasmids. Forty-eight hours after transfection, cell lysates were prepared and analyzed by immunoblotting. As shown in Fig. 5A, forced expression of either ACER2 or p53 alone caused a remarkable induction of autophagy marker LC3-II. It has been well known that mTOR/Akt pathway plays an inhibitory role in autophagy^18^,^30^. Consistently, the amounts of phosphor-mTOR and phosphor-Akt, which suppress autophagy, were decreased in H1299 cells expressing either ACER2 or p53, strongly suggesting that ACER2 has an ability to promote autophagy at least in part through the inhibition of mTOR-Akt pathway. Under the same experimental conditions, transfected cells were analyzed by flow cytometry for apoptosis. As shown in Fig. 5B, overexpression of either ACER2 or p53 alone caused a remarkable increase in PI^+^/AnexinV^+^ apoptotic population, suggesting that, like p53, ACER2 has an ability to induce apoptosis. Notably, co-expression of ACER2 with p53 resulted in a much more larger PI^+^/AnexinV^+^ apoptotic population than ACER2 or p53 alone, indicating that ACER2 augments p53-dependent apoptosis.

In support of the results obtained from immunoblotting, electron microscopic analysis demonstrated a significant accumulation of the double-membraned autophagic vesicles (autophagosomes) in cells transfected with either ACER2 or p53 expression plasmid (Fig. 5C). We then examined the cellular distribution of LC3. Once autophagosomes appear, the diffused cytoplasmic form of LC3, LC3-I, is conjugated with lipid to form LC3-II, which is associated with autophagosome membranes, and thus can be visualized in small puncta. Indirect immunofluorescence staining revealed that overexpression of either ACER2 or p53 causes a marked increase in number of LC3 puncta (Fig. 5D, Supplementary Fig. S1). Notably again, co-expression of ACER2 with p53 resulted in much more double-membraned autophagic vesicles (Fig. 5C) and LC3 puncta (Fig. 5D, Supplementary Fig. S1), and much less phosphorylated mTOR and Akt (Fig. 5A), than p53 or ACER2 alone, suggesting that ACER2 augments p53-dependent autophagy.

### ACER2 contributes to DNA damage-induced autophagy and apoptosis

**Figure 6:**
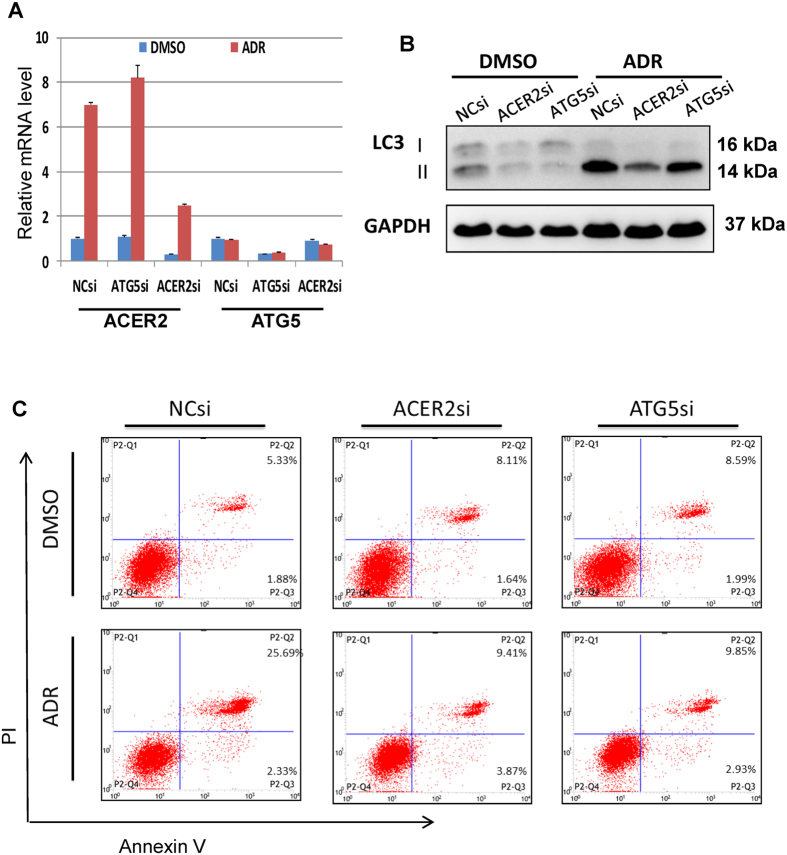
ACER2 and ATG5 are required for DNA damage-induced apoptosis and autophagy. A549 cells were transiently transfected with control siRNA (NCsi), siRNA against ACER2 (ACER2si) or with ATG5 siRNA (ATG5si). Twenty-four hours after transfection, cells were exposed to 2 μM of ADR or left untreated. Thirty-six hours after treatment, cells were subjected to qPCR analysis (**A**), immunoblot analysis (**B**), and flow cytometric analysis (**C**).

**Figure 7:**
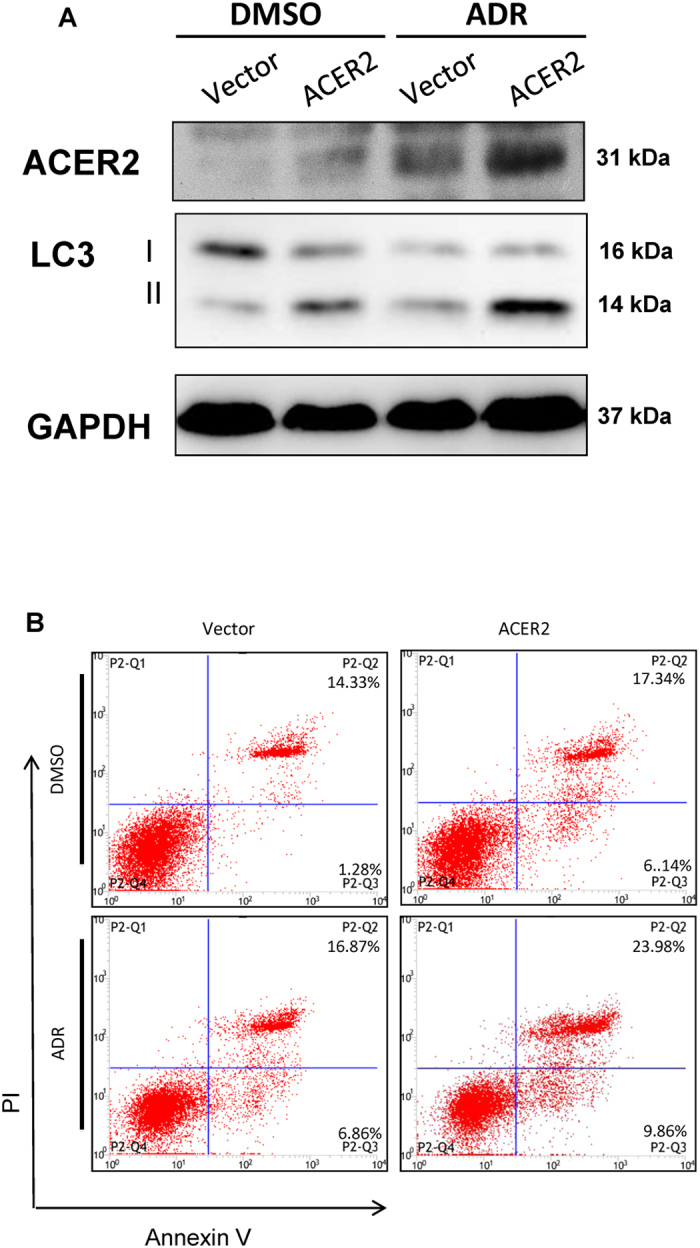
ACER2 enhances DNA damage-induced apoptosis and autophagy. A549 cells were transiently transfected with the empty plasmid or with ACER2 expression plasmid. Twenty-four hours after transfection, cells were incubated in the presence or absence of 2 μM of ADR. Thirty-six hours after treatment, cells were subjected to immunoblot analysis (**A**) and flow cytometric analysis (**B**).

Next, we examined whether ACER2 induction could be required for p53-mediated autophogy and apoptosis following DNA damage. To this end, we have employed siRNA-mediated knockdown of ACER2 in A549 cells bearing wild-type p53. Twenty-four hours after siRNA transfection, cells were treated with DMSO or with ADR (2 μM). Thirty-six hours after treatment, total RNA was isolated and analyzed by qRT-PCR. As shown in Fig. 6A, silencing of ACER2 was successful. Under the same experimental conditions, cell lysates were prepared and analyzed for LC3-II by immunoblotting. As shown in Fig. 6B, ADR-mediated up-regulation of LC3-II was attenuated by ACER2 depletion. FACS analysis revealed that silencing of ACER2 reduces number of PI^+^/AnnexinV^+^ apoptotic cells in response to ADR as compared to cells transfected with control siRNA (Fig. 6C). Consistent with these observations, enforced expression of ACER2 in H1299 cells led to an increase in number of autophagic and apoptotic cells in response to ADR as compared with that of control cells (Fig. 7A and B). Taken together, these results strongly suggest that ACER2 plays an important role in the regulation of p53-induced autophagy and apoptosis in response to DNA damage.

We then examined the effects of ACER2-induced autophagy on apoptosis under our experimental conditions. To this end, we have employed specific siRNA to knockdown the essential autophagy gene ATG5 in A549 cells (Fig. 6A). The results revealed that siRNAs-mediated ATG5 silencing reduces ADR-mediated up-regulation of LC3-II and number of PI^+^/AnnexinV^+^ apoptotic cells in response to ADR as compared to cells transfected with control siRNA (Fig. 6B and C). Of note, ATG5 depletion did not affect ADR-induced ACER2 up-regulation as compared with the cells transfected with control siRNA (Fig. 6A). Thus, these data suggest that ACER2-mediated autophagy facilitates the subsequent apoptotic cell death following DNA damage.

### ACER2 induces apoptosis and autophagy by inducing ROS generation

**Figure 8:**
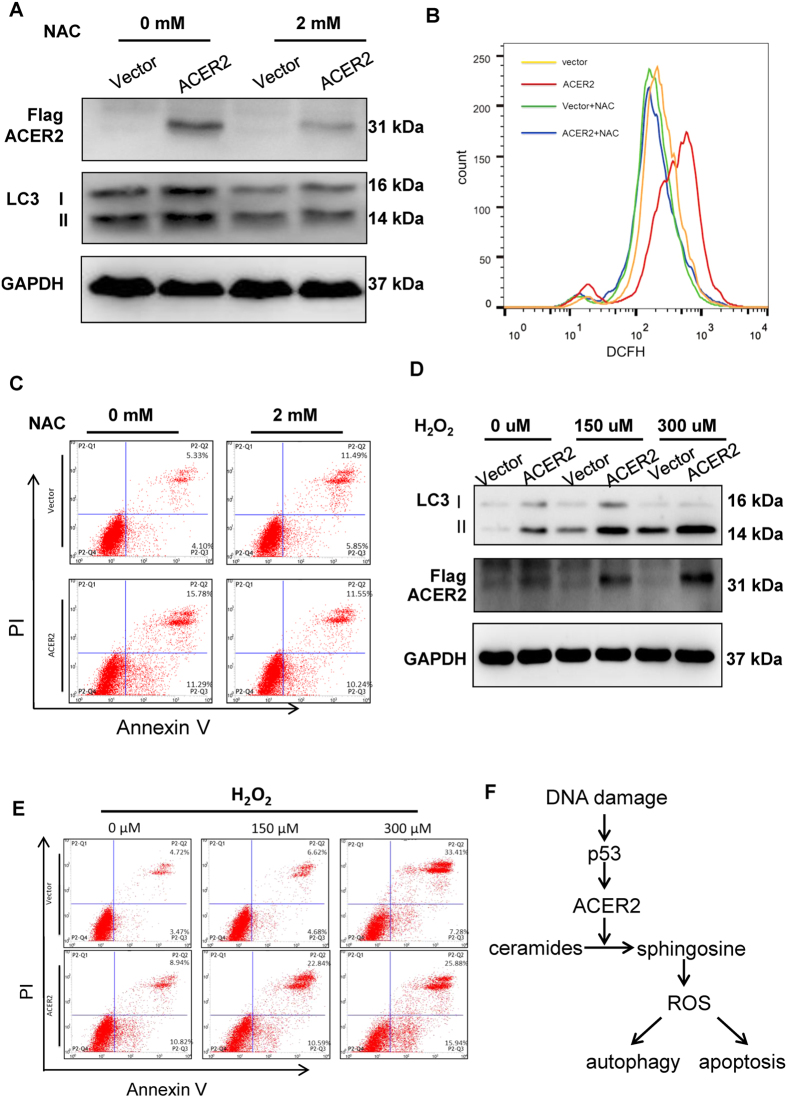
ACER2 induces apoptosis and autophagy though enhancing ROS generation. (**A**–**C**) ACER2-mediated apoptosis and autophagy is prohibited by NAC. H1299 cells were pre-treated with 2 mM N-acetyl-cysteine (NAC) for 2 h, and then transiently transfected with the empty plasmid or with ACER2 expression plasmid. Forty-eight hours after transfection, cells were processed for immunoblot assay (**A**), the flow cytometric analysis for ROS (**B**) and the flow cytometric analysis for apoptosis (**C**). (**D**,**E**) H_2_O_2_ treatment augments ACER2-mediated apoptosis and autophagy. H1299 cells were transiently transfected with the empty plasmid or with ACER2 expression plasmid. Twenty-four hours after transfection, cells were treated with the indicated concentrations of H_2_O_2_ or left untreated. Forty-eight hours after H_2_O_2_ treatment, cells were subjected to immunoblot analysis (**D**) and the flow cytometric analysis for ROS (**E**). (**F**) Schematic representation of p53/ACER2-dependent apoptosis and autophagy. DNA damage causes p53 activation, which subsequently transactivates ACER2. ACER2 enhances sphingosine level and also stimulates the generation of ROS, leading to the induction of autophagy and apoptosis.

Previous studies demonstrated that overexpression of ACER2 induces the generation of its bioactive product, sphingosine, which leads to the production of reactive oxygen species (ROS)^28^,^29^,^31^,^32^,^33^. In addition, it has been well known that ROS potentiates apoptosis and autophagy^34^,^35^. Therefore, we sought to examine whether ACER2 overexpression could augment apoptosis and autophagy by increasing ROS levels. H1299 cells were treated with NAC or left untreated. Two hours after treatment, cells were transfected with the empty plasmid or with the expression plasmid for ACER2. Forty-eight hours after transfection, cells were analyzed by immunoblotting or by flow cytometry. As shown in Fig. 8A – C, ACER2 overexpression resulted in a dramatic increase in ROS level accompanied by the induction of apoptosis and autophogy. However, treatment of cells with ROS scavenger, N-acetyl-cysteine (NAC), ACER2 overexpression-mediated apoptosis and autophagy were significantly impaired. In contrast to the addition of NAC, the exogenous H_2_O_2_ enhanced ACER2 overexpression-mediated apoptosis and autophagy (Fig. 8D and E). Together, these results suggest that ACER2 induces apoptosis and autophagy through the production of ROS.

## Discussion

In addition to its well-established role in carbohydrate and amino acid metabolisms, it has recently been shown that tumor suppressor p53 is implicated also in the regulation of sphingosine metabolism^20^,^36^. Numerous studies revealed that cellular stresses such as DNA damage significantly increase the expression levels of bioactive sphingolipids including ceramides^20^,^37^,^38^,^39^,^40^,^41^,^42^,^43^,^44^, sphingosine^45^ and sphingosine-1-phosphate^46^. In mammalian cells, there are at least 33 distinct enzymes involved in sphingolipid metabolism^28^. Of note, systemic analysis on DNA damage-mediated expression of major sphingolipid-metabolizing enzymes demonstrated that ADR treatment causes a marked increase in ACER2 expression level, whereas the expression levels of the other sphingolipid-metabolizing enzymes remain unchanged or moderately up-regulated in response to ADR^28^. Additional analysis clearly showed that ACER2 promotes ADR-dependent apoptosis through the generation of sphingosine and ROS^28^. These observations strongly suggest that, among sphingolipid metabolizing enzymes, ACER2 contributes to the proper DNA damage response. Intriguingly, our present studies have demonstrated that p53 directly transactivates ACER2, which subsequently induces autophagy and apoptosis in response to DNA damage. Therefore, it is highly likely that ACER2 is transactivated by p53 in response to DNA damage, and contributes to DNA damage-mediated induction of autophagy and apoptosis. With these in mind, ACER2 might be a novel molecular target for cancer therapy.

It has been shown that sphingosine level is elevated in response to DNA damage such as ADR in cancer cells carrying wild-type p53 including MCF7, HCT116 and A549 cells^28^,^45^. From our present findings showing that p53 directly up-regulates ACER2 which catalyzes the hydrolysis of ceramides to generate sphingosines, it is conceivable that p53 might affect the intracellular concentration and/or distribution of sphingosine through the transactivation of ACER2. To confirm this issue, further experiments should be required.

Our present results have also demonstrated that ACER2 is sufficient to induce apoptosis. Consistent with our findings, Xu *et al*. described that DNA damage-dependent up-regulation of ACER2 contributes to the induction of apoptosis through the production of sphingosine and ROS^28^. Although several lines of evidence indicate that sphingosine and its analogs stimulate the production of ROS and thereby inducing apoptosis^31^,^32^,^33^,^47^, it remains elusive how ACER2-mediated generation of sphingosine could enhance the cellular ROS level. Notably, Zigdon *et al*. found that an accumulation of dihrdrosphingosine, a saturated analog of sphingosine, arising from a deficiency of ceramide synthase 2 (CerS2) leads to ROS generation through the inhibition of the mitochondrial respiratory chain^48^. *In vitro* experiments demonstrated that sphingosine and sphinganine directly inhibit complex IV activity of the isolated mitochondria^48^. Therefore, further studies should be required to adequately address whether this could be the case.

To our knowledge, we have demonstrated for the first time that ACER2 also regulates autophagy through the generation of ROS. Autophagy and apoptosis have been generally accepted to be evolutionarily conserved cellular processes to determine cell fate in response to cellular stresses including DNA damage^18^,^49^,^50^. Based on our present results, DNA damage-mediated apoptosis was significantly impaired in ATG5- or ACER2-depleted cells. Thus, it is possible that ACER2 contributes to the induction of not only apoptosis but also autophagy. Previously, Young *et al*. described the sphingolipids-mediated crosstalk between apoptosis and autophagy^18^. According to their results, SKI-I, a pansphingosine kinase inhibitor, potentiated apoptosis accompanied by the induction of autophagy^18^. Since sphingosine kinase inhibitor resulted in an accumulation of sphingosine, it is necessary to further investigate whether ACER2 could participate in sphingosine-induced autophagy and apoptosis.

Notably, the induction of autophagy by sphingosine has not yet been demonstrated^18^. Based on our present findings, it is conceivable that sphingosine, a product of ACER2, could induce autophagy through increasing ROS generation. Further study should be required to adequately address this issue. In addition, it has been shown that ceramide, a substrate of ACER2, regulates both apoptosis and autophagy, and also directly inhibits mitochondrial complex III to generate ROS^18^. However, the functional importance of ROS in ceramide-mediated autophagy and apoptosis has not yet been fully clarified and thus merits further study.

In summary, our present study not only identified the critical sphingolipid metabolizing enzyme ACER2 as a novel direct target of p53, but also demonstrated a novel role of ACER2 in the regulation of autophogy as well as apoptosis though the generation of sphingosine and ROS. Based on our current findings, further extensive studies on the function of ACER2 and sphingosine might provide a clue to develop a promising strategy for cancer therapy.

## Methods

### Reagents and cell lines

Hydrogen peroxide (H_2_O_2_), adriamycin hydrochloride (ADR), N-acetyl-cysteine (NAC), dimethyl sulfoxide (DMSO) and 4′,6-Diamidino-2-phenylindole dihydrochloride (DAPI) were purchased from Sigma-Aldrich (China). Human lung cancer H1299 and A549 cells were maintained in a humidified atmosphere containing 5% CO_2_ at 37 °C in RPMI 1640 medium (H1299) or in DMEM (A549) supplemented with 100 units/ml of penicillin, 100 mg/ml of streptomycin and 10% (vol/vol) heat-inactivated FBS (Invitrogen).

### Rapid amplification of cDNA ends (RACE)

5′-RACE analysis was carried out using SMART^TM^ RACE cDNA amplification kit (Clontech) as described previously^51^. Briefly, human normal testis total RNA (Ambion) was reverse-transcribed to generate first-strand cDNA with the incorporation of an adaptor onto its 5′ end. Two distinct ACER2 gene-specific primers (GSP1 and GSP2) corresponding to its second and fourth exons were designed and synthesized. The primer sequences are listed in Supplementary Table S1. Primary and nested PCR were conducted by using AP/GSP2 and NUP/GSP1, respectively. The resultant nested PCR products were cloned and sequenced.

### Luciferase reporter constructs and reporter assays

The putative promoter region of ACER2 was obtained by PCR-based amplification and cloned into pGL3-basic vector to generate ACER2-P1285. A series of deletion mutants were constructed using Site-Directed Mutagenesis kit (TOYOBO, Japan) according to the manufacturer’s instructions. Luciferase reporter constructs containing the indicated point mutation(s) at the potential p53-binding sites were also generated by using Site-Directed Mutagenesis kit. The sequences of primers used are listed in Supplementary Table S2. All of the reporter constructs were validated by direct sequencing.

For luciferase reporter analysis, cells were seeded in triplicate into 12-well plates and co-transfected with the indicated reporter vectors, pRL-TK vector (Promega) encoding Renilla luciferase together with the empty vector (pcDNA3) or with pcDNA3-Flag-p53 expression vector using Lipofectamine 2000 reagent (Invitrogen). Forty-eight hours after transfection, cells were lysed and their luciferase activities were measured using Dual-Luciferase assay system (Promega) as described previously^51^.

### Construction of the expression plasmids and transient transfection

The entire coding region of human ACER2 was amplified by PCR and inserted into Eco R I/Xho l sites of the mammalian expression plasmid pcDNA3.0-Flag. The resultant expression plasmid was verified by sequencing and named as pcDNA-Flag-ACER2. The mutant p53 expression plasmid, pcDNA-p53 (R175H), was kindly provided by Dr. Giovanni Blandino. For transient transfection, cells were seeded at a density of 1 × 10^5^ cells/12-well tissue culture plate or 3.5 × 10^5^ cells/6-well tissue culture plate and incubated overnight. Cells were then transiently transfected with the indicated plasmids using Lipofectamine 2000 reagent (Invitrogen) according to the manufacturer’s protocols.

### siRNA synthesis and transfection

The negative control siRNA and siRNAs against ACER2 and ATG5 were chemically synthesized by Shanghai GenePharma (Shanghai, China). The sequences of siRNAs used are listed in Supplementary Table S1. siRNAs were transfected into the indicated cells using Lipofectamine RNAiMAX reagent (invitrogen) according to the manufacturer’s instructions.

### RNA isolation and RT-PCR

Total RNA isolation and quantitative RT-PCR were conducted as described previously^52^. The sequences of the primers for PCR are shown in Supplementary Table S1.

### Immunoblot analysis

Immunoblot analysis was performed as described previously with slight modifications^52^. In brief, cells were lysed in 2 × SDS sample buffer (for the detection of ACER2 protein in Golgi membranes) or in RIPA buffer (for the detection of the other proteins) supplemented with protease inhibitor PMSF (Beyotime, Jiangsu, China). Total proteins were quantified using the bicinchoninic acid (BCA) protein assay kit (Thermo Scientific, Beijing, China). The antibodies used in the present study were shown in Supplementary Table S3. The blots were visualized by the enhanced chemiluminescence (ECL, Bio-Rad Laboratories, USA).

### Chromatin immunoprecipitation (ChIP)

ChIP was performed using EZ ChIP^TM^ Chromatin Immunoprecipitation kit (Upstate, Lake Placid, NY) as described previously^51^. The sequences of the primers and the antibodies used were provided in Supplementary Tables S1 and S3, respectively. qPCR data for ChIP were calculated and shown as % of recovered immunoprecipitated DNA relative to the Input DNA, i.e. % Input = 100 × 2^−∆Ct^, where ∆Ct = (Ct [ChIP] - (Ct [Input] - Log_2_100)).

### Indirect immunofluorescence staining

Cells were seeded on glass cover slips coated with poly-lysine in a 24-well culture plate, and then transfected with the indicated plasmids. Forty-eight hours after transfection, cells were fixed in 4% paraformaldehyde in 1 × PBS, permeabilized with 0.05% Triton X-100 at room temperature for 15 min, and blocked with 0.5% BSA and 10% goat serum for 1 h at 37 °C. After blocking, cells were incubated with anti-LC3 antibody for overnight at 4 °C followed by the incubation with secondary antibody conjugated with fluorescein isothiocyanate (FITC) for 2 h. After washing in 1 × PBS, cells were incubated with 4,6-diamidino-2-phenylindole (DAPI), and cover slips were mounted onto glass slides by using ProLong Gold Antifade Reagent. Cells were observed under a confocal microscope.

### Staining autophagosomes with GFP-LC3

Cells seeded on glass cover slips coated with poly-lysine in a 24-well culture plate were co-transfected with 100 ng of GFP-LC3 together with 200 ng of pcDNA3.0-Flag empty vector, pcDNA3.0-Flag-ACER2 or with pcDNA3.0-Flag-p53 using Lipofectamine 2000 (Invitrogen). Forty-eight hours after transfection, the fluorescence of GFP-LC3 was observed under a confocal microscope. Cell nuclei and the exogenous Flag-ACER2/Flag-p53 were stained with DAPI and anti-Flag antibody, respectively.

### Flow cytometric analysis for apoptosis

Annexin V-FITC apoptosis detection kit (BD Pharmingen) was used to detect apoptotic activity according to the manufacturer’s instructions with slight modifications. Briefly, cells were harvested, washed twice in ice-cold 1 × PBS and resuspended in binding buffer. Annexin V-FITC and propidium iodide (PI) were then added to the reaction mixture, and incubated for 15 min at room temperature in the dark. Cells were then analyzed for apoptosis by flow cytometry.

### Flow cytometric analysis for ROS

Reactive oxygen species was detected by cell-permeable fluorogenic probe 2′, 7′-dichlorodihydrofluorescin diacetate (DCFH-DA) according to the manufacturer’s instructions. Briefly, cells were washed in 1 × PBS and incubated with DCFH-DA-containing medium at 37 °C for 30 min. Cells were then washed in 1 × PBS and subjected to DCF fluorescent quantification using flow cytometry.

### Transmission electron microscopy (TEM)

Cells were pre-fixed in 4% glutaraldehyde for 1.5 h at 4 °C and fixed in 1% OsO4. After dehydration and embedding, ultrathin sections were prepared with a Reichert-Jung Ultracut Ultramicrotome (Leica, Vienna, Austria). Images were observed under a H7500 transmission electron microscope (Hitachi Ltd, Tokyo, Japan).

### DNA sequence alignment and database analysis

ACER2 mRNA and genomic sequences were extracted from GeneBank and UCSC database (https://genome.ucsc.edu/). The 5′-RACE sequences were aligned with ACER2 genomic sequences to verify gene identity, exon usage and location of the transcription start sites. The potential transcription factor-binding sites within ACER2 promoter were predicted by MatInspector professional (http://www.genomatix.de/) and TFSEARCH software. Sequence alignment of the 5′-upstream regions of ACER2 across species was performed using ClustalW2 algorithm at European Bioinformatics Institute (http://www.ebi.ac.uk/Tools/msa/).

## Additional Information

**How to cite this article**: Wang, Y. *et al*. Alkaline ceramidase 2 is a novel direct target of p53 and induces autophagy and apoptosis through ROS generation. *Sci. Rep.* **7**, 44573; doi: 10.1038/srep44573 (2017).

**Publisher's note:** Springer Nature remains neutral with regard to jurisdictional claims in published maps and institutional affiliations.

## Supplementary Material

Supplementary Information
