# Supplementary material for: Alkaline ceramidase 2 is a novel direct target of p53 and induces autophagy and apoptosis through ROS generation
Source: Sci Rep. 2017 Mar 15;7:44573. doi: 10.1038/srep44573 (PMC5353723; doi:10.1038/srep44573)
Supplement: Supplementary Information [file srep44573-s1.pdf]

Supplementary Information for

Alkaline ceramidase 2 is a novel direct target of p53 and induces autophagy and apoptosis through ROS generation

Yitao Wang[1,2#], Chunxue Zhang[1,2#], Yuelei Jin[1,2], Sen Wang[1,2], Qing He[1,2], Zhu Liu[1,2], Qing Ai[1,2], Yunlong Lei [1,2], Yi Li [1,2],Fangzhou Song[1,2], and Youquan Bu[1,2*]

[1] Department of Biochemistry and Molecular Biology, and [2] Molecular Medicine and Cancer Research Center, Chongqing Medical University, Chongqing 400016, China

[#] These authors contributed equally to this work.

* To whom all correspondence should be addressed. Youquan Bu. Department of Biochemistry and Molecular Biology, ChongQing Medical University. 1# Yixueyuan Road, Yuzhong District, ChongQing 400016 China. Tel: +86-23-68485991; E-mail: buyqcn@cqmu.edu.cn, buyqcn@aliyun.com

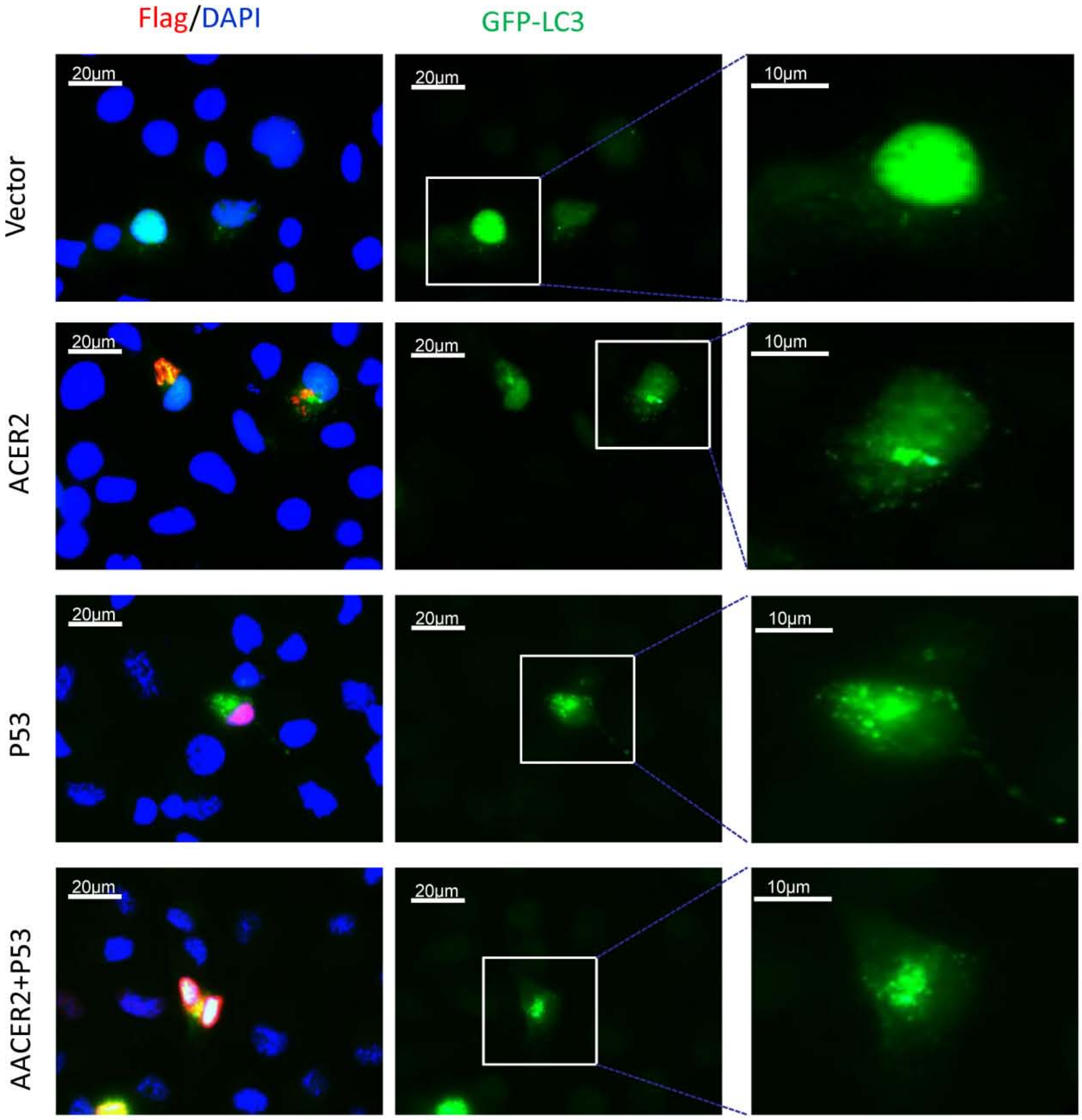

Supplementary Figure 1. ACER2 overexpression autophagy induces autophagosome formation.

H1299 cells were transiently transfected with empty vetor, ACER2 and/or p53 expression plasmids together with GFP-LC3 expressing plasmid. Forty-eight hours after transfection, cells were assayed for the appearance of autophagosomes with the formation of GFP-LC3 puncta by confocal microscopy. Ectopic Flag-ACER2 and Flag-p53 expression were determined by indirect immunofluorescence assay with Flag antibodies (red). Cell nuclei were stained with DAPI (blue).

Supplementary Table 1. Oligonucleotides used in the present study

Primers used for RACE analysis

Primer name	Primer sequences
GSP1-325	5'-TGCTGATCGTGTTGTAGAACTCGGCG-3'
GSP2-601	5'-CACTGACCACCACCTTGAACCTACCC-3'

Primers used for RT-PCR analysis

Gene name	Primer sequence
GAPDH	F833: 5'-ACCTGACCTGCCGTCTAGAA-3'
	R1060: 5'-TCCACCACCCTGTTGCTGTA-3'
ACER2	F562: 5'-GTAGGTTCAAGGTGGTGGTCAG-3'
	R680: 5'-CACACGCATGTTGTCACACCT-3'
P21	F990: 5'-ATGAAATTCACCCCCTTTCC-3'
	R1163: 5'-CCCTAGGCTGTGCTCACTT-3'
Bax	F112: 5'-GAGCTGCAGAGGATGATTGC-3'
	R313: 5'-CCAATGTCCAGCCCATGATG-3'
MDM2	F659: 5'-AGCAGGAATCATCGGACTCA-3'
	R877: 5'-TGTGGCGTTTTCTTTGTCGT-3'
P53	Forward: 5'-GTTCCGAGAGCTGAATGAGG-3'
	Reverse: 5'-TCTGAGTCAGGCCCTTCTGT-3'

Primers used for ChIP analysis

Primer name	Primer sequence	Amplified region
ACER2-ChIP-F2541	5'-GGAACCAGGGGTCTGTGTCA-3'	
ACER2-ChIP-R2684	5'-AGGTATTCAGGGCAAAAAAGC-3'	(+459 ~ +602 bp)
ACER2-ChIP-F2763	5'-ACCCCCAAGAACATGCCTATAA-3'	
ACER2-ChIP-R2914	5'-AGACTTCAGAACTGGAGAAAGCA-3'	(+681 ~ +832 bp)
P21-ChIP-F	5'-GTGGCTCTGATTGGCTTTCTG-3'	
P21-ChIP-R	5'-CTGAAAACAGGCAGCCCAAG-3'	

siRNAs

Name	Sequences
Negative control siRNA	Sense: 5'-UUCUCCGAACGUGUCACGUUU-3'
	Antisense: 5'-AAACGUGACACGUUCGGAGAA-3'
ACER2 siRNA	Sense: 5'-GCCUGCCAUCAACAACAUCTT-3'
	Antisense: 5'-GAUGUUGUUGAUGGCAGGCTT-3'
ATG5 siRNA	Sense: 5'-CCAACUUGUUUCACGCUAUTT-3'
	Antisense: 5'- AUAGCGUGAAACAAGUUGGTT-3'

Supplementary Table 2. Luciferase reporter construction

Constructs	Methods	Primers and/or Enzymes used
P1258(-470/+815)	PCR based cloning	F(1613): 5'-ACC GAG CTC ACT TCT CTG GTG TAA GCA CTG GC-3'*Sac* I
		R(2897): 5'-CTA GCT AGC AAA GCA CAG TCC TGG GGG AGT TG-3'*Nhe* I
		Template: human genomic DNA
P800 (+15/+815)	PCR based blunting	F(2097):5'-CCGCAGCAGCTCTGGGCTCTTCTCA-3'
		pGL3b-mutationR:5'-GGTACCTATCGA TAG AGA AAT GTT CTG GC-3'
		Template: P1258(-470/+815)
P676(-140/+815)	PCR based blunting	F(2222):5'-GACAACTACACCATCGTGCCTGCTA-3'
		pGL3b-mutationR:5'-GGTACCTATCGA TAG AGA AAT GTT CTG GC-3'
		Template: P1258(-470/+815)
P397 (+419/+815)	PCR based blunting	F(2501): 5'-TGTGATG GTCCTATTGTTCTTGTTTG-3'
		pGL3b-mutationR:5'-GGTACCTATCGA TAG AGA AAT GTT CTG GC-3'
		Template: P1258(-470/+815)
P304 (+410/+443)	PCR based blunting	pGL3b-mutationF: 5'- GCTAGCCCGGGCTCGAGATCTGCGATCT -3'
		R(2525): 5'- CAAACAAGACAATAGGACCATCACA -3'
		Template: P676(-140/+815)
P223 (+140/+362)	PCR based blunting	pGL3b-mutationF: 5'- GCTAGCCCGGGCTCGAGATCTGCGATCT -3'
		R(2444): 5'-GGAGGACAGATGGAGAACTGAAAGG-3
		' Template: P676(-140/+815)
P136 (+140/+275)	PCR based blunting	pGL3b-mutationF: 5'- GCTAGCCCGGGCTCGAGATCTGCGATCT -3'
		R(2357): 5'- AGAGACCCACGTCCAGCTTCCAGC-3'
		Template: P676(-140/+815)
P397 –P53M1	PCR based mutagenesis	ACER2-p53-m1-F: 5'- AAGTCTCTTCCTCCCCTGGG-3'
		ACER2-p53-m1-R: 5'- GTCCCGG<u>AAGT</u>TTCACCG-3' wt: CAGG
		Template: P397 (+419/+815)
P397 –P53M2	PCR based mutagenesis	ACER2-p53-m2-F: 5'- CCCCACCCCCAAGAACAT-3'
		ACER2-p53-m2-R: 5'- <u>AAAT</u>TCTGCCTGGTGGGAG-3' wt: CAAG
		Template: P397 (+419/+815)
P397 –P53M1+2	PCR based mutagenesis	ACER2-p53-m2-F: 5'- CCCCACCCCCAAGAACAT -3'
		ACER2-p53-m2-F: 5'- <u>AAAT</u>TCTGCCTGGTGGGAG-3' wt: CAAG
		Template: P397 –P53M1

Supplementary Table 3. Antibodies used in the present study

Protein name	***Maneufacture (cat. number)***	***Applications (working dilution)***	***Website Link***
GAPDH	Xianzhi Bio(AB-P-R 001)	IB(1:5000)	http://www.goodhere.com/showproduct.asp?id=320&classid=34&nid=2
LC3A/B	CST(12741)	IB(1:2000) IF(1:200)	https://www.cellsignal.com/products/primary-antibodies/lc3a-b-d3u4c-xp-rabbit-mab/12741?N=4294956287&Ntt=LC3&fromPage=plp
Flag	sigma (F1804)	IB(1:2000) IF(1:200)	http://www.sigmaaldrich.com/catalog/product/sigma/f1804?lang=zh&region=CN
ACER2	sigma (HPA014092)	IB(1:500)	http://www.sigmaaldrich.com/catalog/product/sigma/hpa014092?lang=zh&region=CN
mTOR	CST(2983)	IB(1:2000)	https://www.cellsignal.com/products/primary-antibodies/mtor-7c10-rabbit-mab/2983?N=4294956287&Ntt=mTOR&fromPage=plp
p-mTOR	CST(5536)	IB(1:2000)	https://www.cellsignal.com/products/primary-antibodies/phospho-mtor-ser2448-d9c2-xp-rabbit-mab/5536?N=4294956287&Ntt=P+mTOR&fromPage=plp
Akt	Ruiying Bio(RLT0178)	IB(1:1000)	http://rlgene.com/showproduct.asp?/1_815
p-Akt	Ruiying Bio(RLP0006)	IB(1:500)	http://rlgene.com/showproduct.asp?/1_439
antiMouse seccondary antibody	Abgent(ASS1007)	IB(1:5000)	http://www.abgent.com/products/ASS1007-Goat-Anti-Mouse-IgGHL-Human-ads-HRP-Secondary-Antibody
antiRabbit seccondary antibody	Abgent(ASS1009)	IB(1:5000)	http://www.abgent.com/products/ASS1009-Goat-Anti-Rabbit-IgGHL-MouseHuman-ads-HRP-Secondary-Antibody
antiMouse seccondary antibody	Thermo(A-11031)	IF(1:1000)	https://www.thermofisher.com/antibody/product/Goat-anti-Mouse-IgG-H-L-Secondary-Antibody-Polyclonal/A-11031
antiRabbit seccondary antibody	Thermo(A-11034)	IF(1:1000)	https://www.thermofisher.com/antibody/product/Goat-anti-Rabbit-IgG-H-L-Secondary-Antibody-Polyclonal/A-11034
p53	Santa Cruz (SC-126X)	ChIP(1:500)	http://www.scbt.com/datasheet-126-p53-do-1-antibody.html
Normal mouse IgG	Milpore (12-271)	ChIP(1:500)	http://www.merckmillipore.com/CN/zh/product/Normal-Mouse-IgG,MM_NF-12-371
